# Melioidosis: misdiagnosed in Nepal

**DOI:** 10.1186/s12879-019-3793-x

**Published:** 2019-02-19

**Authors:** Neha Shrestha, Mahesh Adhikari, Vivek Pant, Suman Baral, Anjan Shrestha, Buddha Basnyat, Sangita Sharma, Jeevan Bahadur Sherchand

**Affiliations:** 1 Department of Microbiology, Tribhuvan University Teaching Hospital (TUTH), Institute of Medicine, Kathmandu, Nepal; 2 Department of Biochemistry, Tribhuvan University Teaching Hospital (TUTH), Institute of Medicine, Kathmandu, Nepal; 3 Department of Medicine, Tribhuvan University Teaching Hospital (TUTH), Institute of Medicine, Kathmandu, Nepal; 4 Department of Pathology, Tribhuvan University Teaching Hospital (TUTH), Institute of Medicine, Kathmandu, Nepal; 5 The Oxford University Clinical Research Unit, Kathmandu, Nepal

**Keywords:** Melioidosis, *Burkholderia pseudomallei*, Diabetes, Abscess, Nepal

## Abstract

**Background:**

Melioidosis is a life-threatening infectious disease that is caused by gram negative bacteria *Burkholderia pseudomallei*. This bacteria occurs as an environmental saprophyte typically in endemic regions of south-east Asia and northern Australia. Therefore, patients with melioidosis are at high risk of being misdiagnosed and/or under-diagnosed in South Asia.

**Case presentation:**

Here, we report two cases of melioidosis from Nepal. Both of them were diabetic male who presented themselves with fever, multiple abscesses and developed sepsis. They were treated with multiple antimicrobial agents including antitubercular drugs before being correctly diagnosed as melioidosis. Consistent with this, both patients were farmer by occupation and also reported travelling to Malaysia in the past. The diagnosis was made consequent to the isolation of *B. pseudomallei* from pus samples. Accordingly, they were managed with intravenous meropenem followed by oral doxycycline and cotrimoxazole.

**Conclusion:**

The case reports raise serious concern over the existing unawareness of melioidosis in Nepal. Both of the cases were left undiagnosed for a long time. Therefore, clinicians need to keep a high index of suspicion while encountering similar cases. Especially diabetic-farmers who present with fever and sepsis and do not respond to antibiotics easily may turn out to be yet another case of melioidosis. Ascertaining the travel history and occupational history is of utmost significance. In addition, the microbiologist should be trained to correctly identify *B. pseudomallei* as it is often confused for other *Burkholderia* species*.* The organism responds only to specific antibiotics; therefore, correct and timely diagnosis becomes crucial for better outcomes.

## Background

Melioidosis is an infectious disease that is potentially acquired by ingestion, inhalation or inoculation of gram-negative bacillus *Burkholderia pseudomallei* [1]. This bacteria is an environmental saprophyte found in soil and stagnant water and is typically endemic to south-east Asia and northern Australia [2, 3]. Nonetheless, melioidosis has been reported from other areas like South Asia as well. Those cases were characterized by travel history or history of exposure to imported animals, soil or even plant [4]. The clinical spectrum of melioidosis is broad, which ranges from subclinical cases to fulminant septicemia with disseminated abscesses especially in immunocompromised patients [5].

## Case presentation

### Case I

**Fig. 1:**
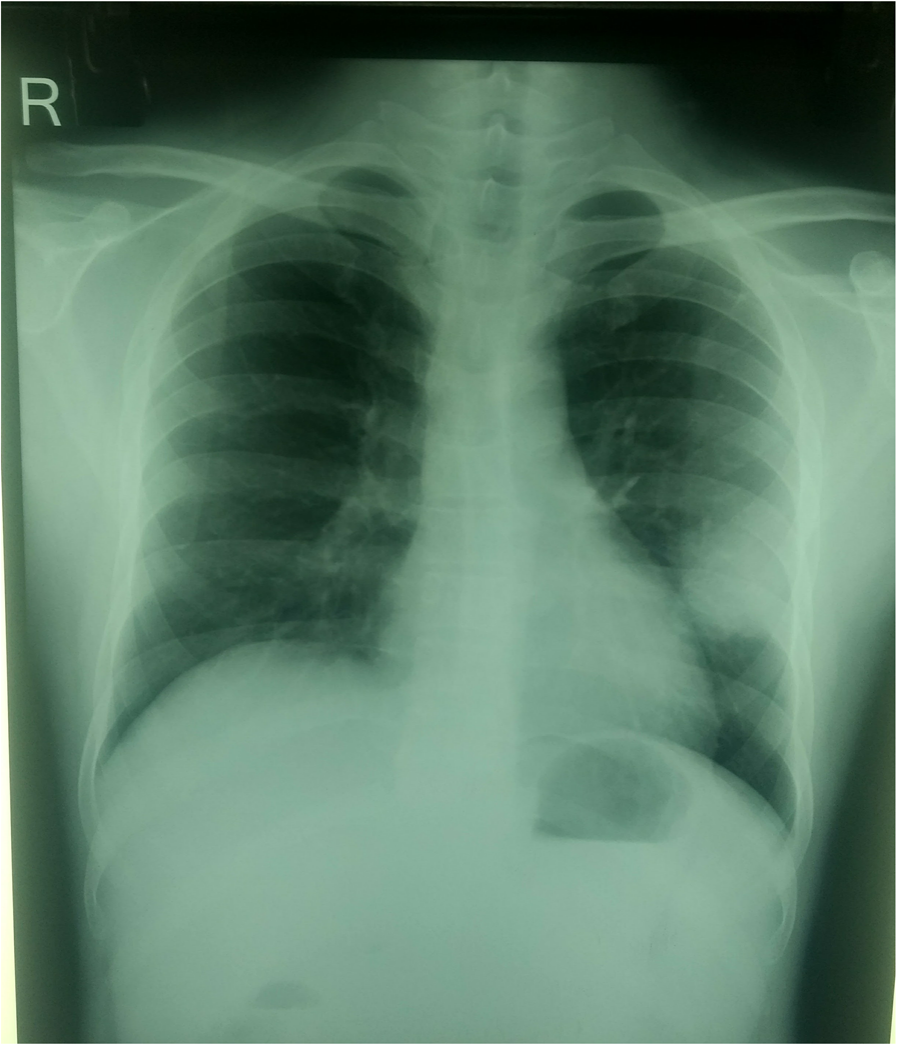
(Case I) Chest X-ray showing cavitary lesion in left lower lobe

A 34-years male was referred to our institute, Tribhuvan University Teaching Hospital. He initially complained of fever, cough and chest pain for last one and half month. The fever was high grade (maximum temperature recorded being 103 °F) associated with chills and rigor. He further complained of anorexia, vomiting, generalized body ache, weight loss and had also developed swelling in his left lower limb (below lateral malleolus) spontaneously. With these complaints, he visited a local hospital where he was diagnosed with diabetes mellitus (random blood sugar- 35.8 mmol/L) and was accordingly managed with insulin. The swelling in his leg was in fact an abscess, which was drained. Interestingly, his chest radiography showed cavitary lesion on left lower lobe (Fig. 1). The subsequent CT scan was indicative of aspergilloma; therefore, he was referred to a higher center.

The patient had tachypnea (respiratory rate-26/min); tachycardia (pulse rate-110/min); hypotension (blood pressure-70/50 mmHg); fever (102 °F) and anemia at the time of his presentation at TUTH. The subsequent auscultation revealed decreased air entry on the left side of his chest with infra-axillary crepitation whereas the palpation showed that the right hypochondriac region was tender but without any organomegaly. His blood examination results were: total leucocyte count- 12,700/mm^3^ with predominant polymorphonuclear leucocyte, hemoglobin- 7.6 g%, random blood sugar- 6.8 mmol/L, urea- 8.9 mmol/L, creatinine-202 μmol/L, sodium- 119.4 mEq/L and potassium- 3.2 mEq/L. Ultrasonography of abdomen revealed multiple small hypoechoic cystic lesions suggestive of abscess in right lobe of liver with minimal ascitic fluid. The patient was immediately admitted into the intensive care unit and a set of drugs like voriconazole, metronidazole and piperacillin-tazobactam were started empirically taking into account his concurrent diagnosis (aspergilloma with pyogenic liver abscess).

**Fig. 2:**
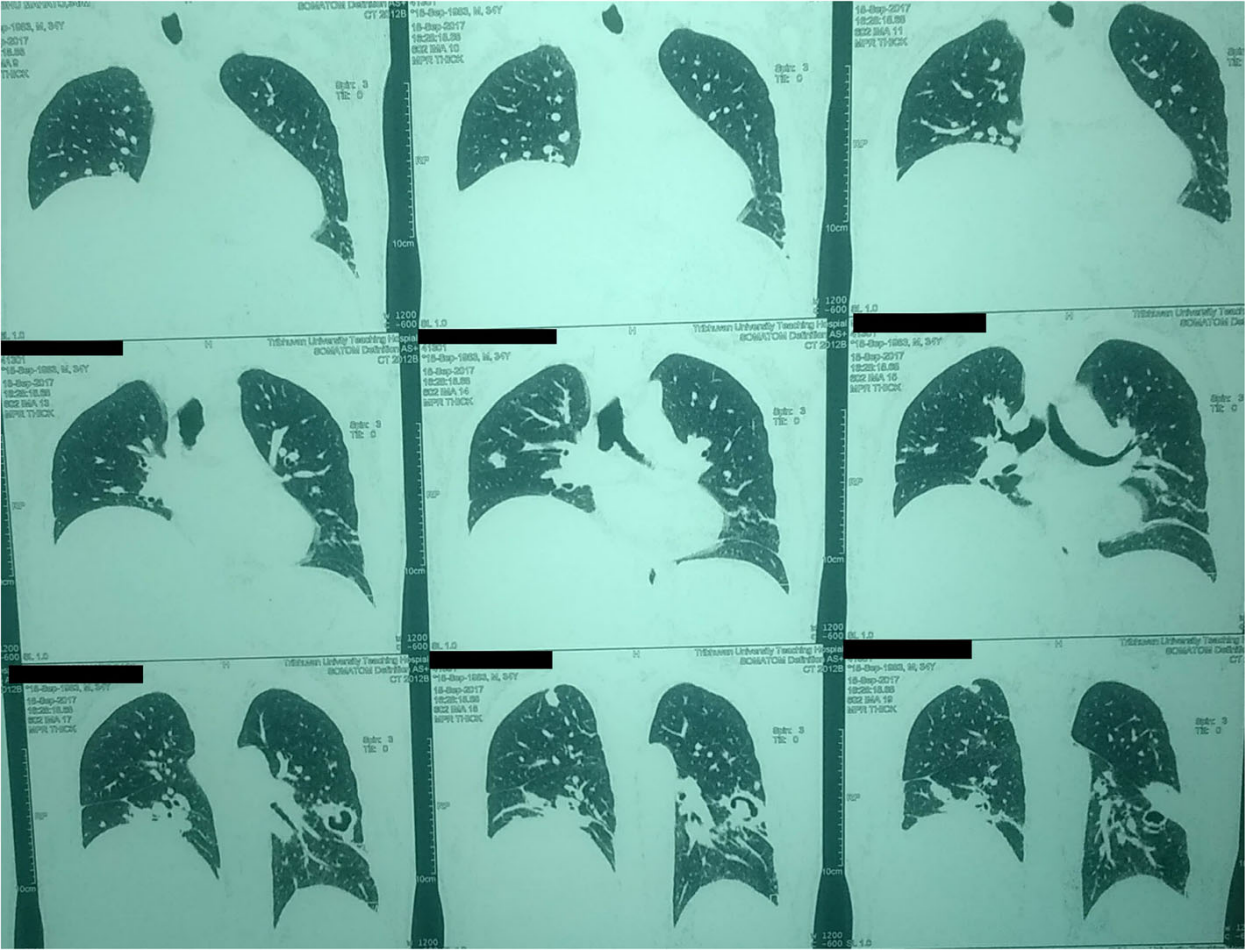
(Case I) HRCT Chest showing multiple thick irregular walled cavities in left upper and lower lobe

Meanwhile, *Klebsiella pneumoniae* was isolated from sputum while urine, blood and wound sample were found to be sterile. In addition, sputum for Acid Fast Bacilli and KOH mount for fungal hyphae turned out negative. Despite antibiotic coverage for nine days, high fever persisted and thus meropenem was started as per the antibiotic sensitivity report of *K. pneumoniae*. Consequently, high resolution CT scan of chest was performed which suggested the lesion to be tuberculosis as multiple cavities were seen in left lobe together with fibrotic changes in upper right lobe (Fig. 2). Accordingly, antitubercular drugs were started owing to patient’s persistent unresponsiveness to wide arrays of antimicrobial agents. However, meropenem was continued as liver abscess was still a mystery.

**Fig. 3:**
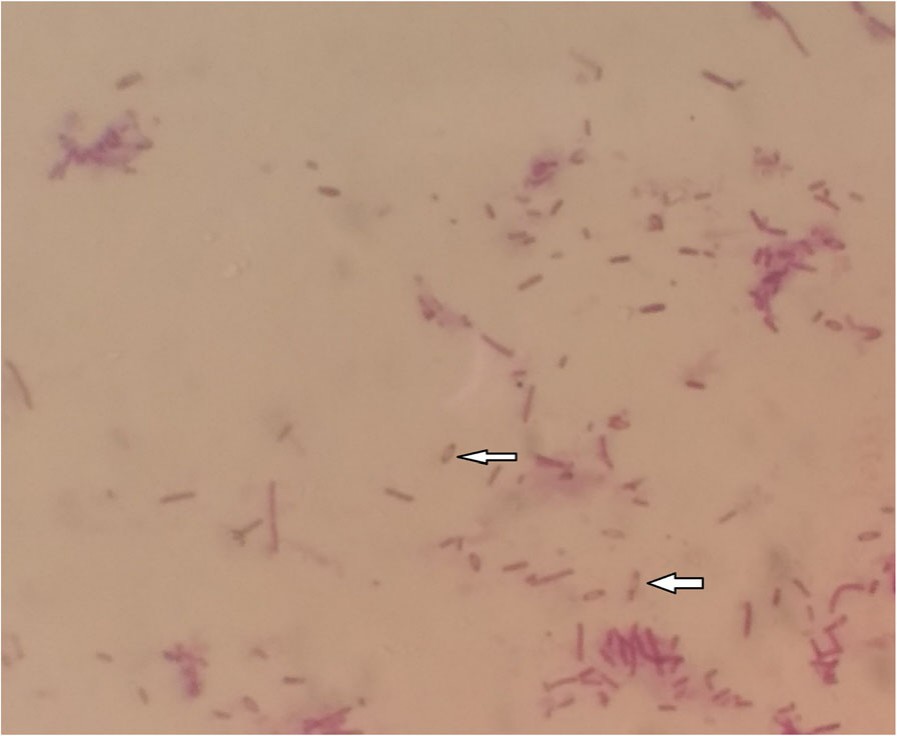
Gram negative bacteria with peculiar Safety pin appearance

**Fig. 4:**
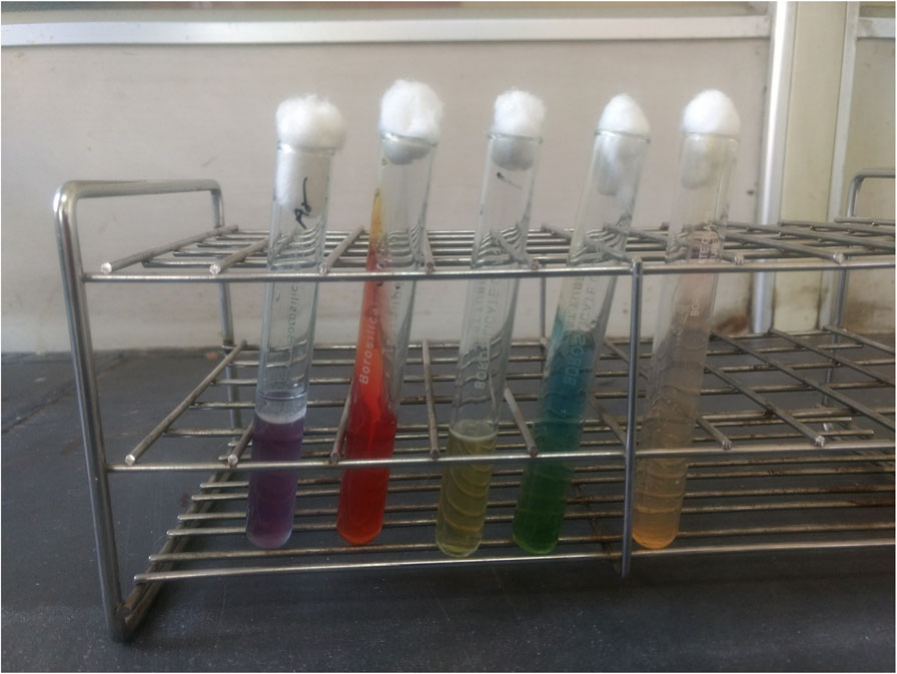
Biochemical reaction from right to left: Arginine decarboxylase test, Triple sugar iron agar, Sulphide Indole Motility agar, Citrate and Urea hydrolysis test

**Fig. 5:**
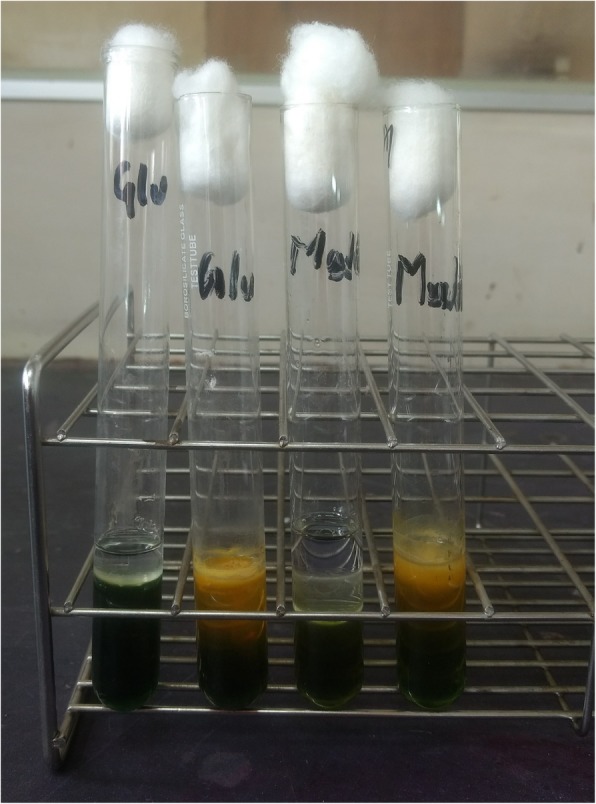
Oxidative utilization of glucose and maltose

**Fig. 6:**
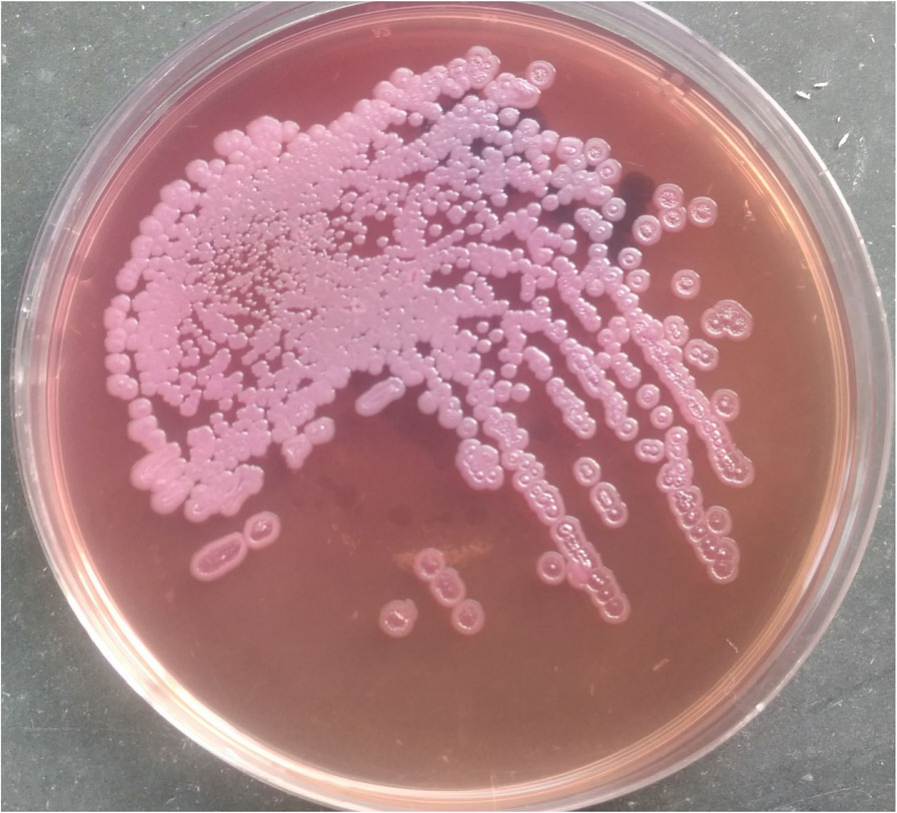
Wrinkled pink colony in MacConkey agar after 48 h of incubation

Thereafter, ultrasonography guided aspiration of liver abscess was planned for diagnostic purpose and frank pus was obtained which was send to microbiology laboratory for further evaluation. On gram staining, the pus sample showed few gram-negative bacilli with plenty of pus cells. The sample was inoculated on blood agar and MacConkey agar which was then incubated at 37 °C. After overnight incubation; large, creamy, smooth colony developed on blood agar and large, smooth pinkish colony was seen in MacConkey agar; gram staining of which revealed gram negative bacilli with typical safety pin (bipolar staining) appearance (Fig. 3). This organism was motile, oxidase and catalase positive. The colony was then processed for various biochemical tests (Fig. 4). The next day, the organism was found to produce arginine dihydrolase and reduce nitrate. In addition, it could utilize glucose and maltose oxidatively (Fig. 5) but could not utilize citrate and hydrolyze urea. Peculiarly, the colony (after 48 h) had turned into wrinkled appearance (Fig. 6) and the organism could grow even at 42 °C. The organism was resistance to polymyxin B, colistin and aminoglycosides (amikacin and gentamicin) whereas sensitive to ceftazidime, meropenem, cotrimoxazole and amoxicillin-clavulanic acid as per the antibiotic susceptibility test.

**Fig. 7:**
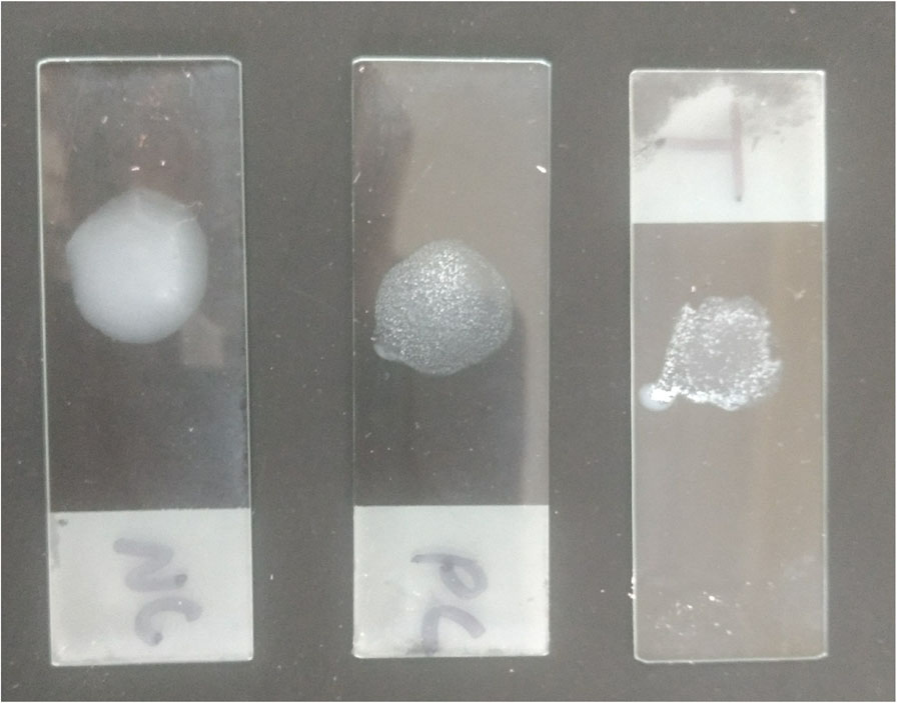
Latex agglutination test positive for *B. pseudomallei*

Notably, the isolate was suggestive of *Burkholderia pseudomallei.* Therefore, commercially available monoclonal antibody based latex agglutination assay for detection of *B. pseudomallei* was carried out from the colony which was also positive (fine agglutinates) (Fig. 7).

Being isolated from Nepal, travel and occupational history was important. In line with this, the patient upon interrogation revealed his travel to Malaysia some seven years ago. There he worked for roughly three years, after which he returned to Nepal and continued farming.

The patient was diagnosed of melioidosis and intravenous meropenem (1 g 8 hourly) was continued for a total of 28 days while antitubercular drugs were withdrawn: in the meantime, patient had also developed ATT induced hepatitis. The patient became afebrile after eleven days of treatment with meropenem and soon his respiratory symptoms improved both clinically and radiologically. After completion of initial phase of therapy, patient was discharged on oral doxycycline (100 mg 12 hourly) and oral cotrimoxazole (960 mg once daily) for three months.

### Case II

A 48 years old diabetic male complained of intermittent high fever associated with chills and rigor, abdominal discomfort and generalized body ache for more than a year. He had visited several hospitals with these complaints and had already been treated with several antimicrobial agents that included antitubercular and antimalarial drugs. However, his symptoms persisted.

He had also visited our hospital six months ago when he was diagnosed as Brucellosis (based on *Brucella abortus* antibody titre > 1:320) with splenic abscess. He was then treated with doxycycline and rifampicin for three weeks. It should be noted that aminoglycoside was not preferred due to deranged renal function test. Eventually, fever subsided and patient remained asymptomatic for three months. Unexpectedly, the patient re-developed high fever and visited our center again. This time he complained of accompanying pain in the right elbow that was consecutive for five days. A thorough examination revealed that he was anemic but his respiratory and gastrointestinal findings were normal. The lateral aspect of right elbow was tender; however, no swelling or redness was noticeable. In addition, blood examination revealed normocytic normochromic anemia, raised inflammatory markers like ESR and C-reactive protein, deranged renal function test, raised random blood sugar (32 mmol/L) and raised Brucella Ab titre (both IgG and IgM). His chest radiography showed infiltration in left upper and middle zone of lung whereas the ultrasonography of abdomen showed splenomegaly. The patient was treated with ceftriaxone and flucloxacillin and his blood sugar level was maintained to normal by intravenous insulin. But fever didn’t subside instead an abscess developed in lateral part of his right elbow which was drained and pus was sent for evaluation in microbiology laboratory.

The patient’s condition had begun to deteriorate after fifth day of admission, which accompanied high fever (5 spikes with maximum 104 °F), tachycardia, tachypnoea and decreased oxygen saturation below 60%. Therefore, he was immediately shifted to intensive care unit and managed.

Meanwhile, the pus sample showed gram negative bipolar bacilli in the gram stain. The organism formed off white wrinkled colony on blood agar and pinkish wrinkled colony on MacConkey agar at 48 h of incubation. Various biochemical tests were performed that suggested the organism to be *Burkholderia pseudomallei* which was susceptible to ceftazidime, meropenem and doxycycline but resistant to amoxicillin with clavulanic acid, polymyxin B, colistin and aminoglycoside.

The patient had traveled to Malaysia ten years ago for employment and stayed there for four years, after which he returned to Nepal and indulged in farming. His travel and occupational history accord with the diagnosis of melioidosis. Thus, the patient was treated with intravenous meropenem for the next 28 days. For eradication phase, oral cotrimoxazole and oral doxycycline was prescribed for three months.

## Discussion

It is well-established that melioidosis poses serious threat to endemic region of south-east Asia and northern Australia [3]. In this context, it is quite alarming that the causative agent has recently been isolated from populous countries like India and China. In fact, multiple cases of melioidosis have already been documented, the primary victim being the local farmers [6, 7]. Much peculiarly, melioidosis has only been reported once in Nepal, which dates back to 2004 AD. The patient hailed from eastern part of Nepal (Dharan). Owing to his travel history to Malaysia, the infection was labelled ‘imported’ [8].

However, in this regard, it should be noteworthy that this disease is still in its infancy and thus remains to be adequately studied in Nepal. The potential burden accompanying melioidosis is yet to be understood. Given the lack of convincing evidence that this disease is solely inherited by travelling to endemic regions, it is tempting to suspect that the source could be intrinsic to Nepal. Fittingly, it is hard to imagine why Nepal would be spared of melioidosis despite south Asia being the hub of this disease [1, 9]. Nowadays, many Nepalese travel abroad to seek employment. In this pursuit, they relocate to country like Malaysia where they are naturally at increased risk of *B. pseudomallei* infection. Taken together, it is difficult to overlook the fact that existing lack of awareness amongst physicians and microbiologists could have resulted into melioidosis being under reported.

Melioidosis is caused by a gram negative bipolar, obligate aerobe *Burkholderia pseudomallei* [10]. The disease has a protean manifestation and is referred to as “remarkable imitator” [11]. Its presentation may vary from inapparent infection, acute localized suppurative infection and acute septicemia to chronic suppurative infection. Pulmonary infection is the most common form of presentation that is likely involved primarily through inhalation or secondarily via hematogenous route [12]. However, virtually any organ like lung, skin, subcutaneous tissue, bones and joints, liver, spleen, bladder, genital organs, brain, pericardium etc. may be involved. Most interestingly, the incubation period can dramatically vary between 2 days to 26 years [13]. In our case, both patients had travelled to Malaysia and had developed the disease many years later. Besides, both were farmers. Therefore, we could not ascertain whether the infection was acquired locally or by travelling to the endemic region.

Risk factors like diabetes mellitus, renal diseases, thalassemia, pulmonary tuberculosis, chronic lung or liver diseases, alcohol abuse and malignancy can contribute to the development of melioidosis; diabetes mellitus being the most common associated factor [1, 14]. Notably, both of the patients in our case were diabetic. Although one of them was newly diagnosed, his condition was presumably longstanding.

Melioidosis is often misdiagnosed as tuberculosis especially in South Asia where the burden of tuberculosis is pretty high [15]. This exposes the patient to unwanted side effects of antitubercular drug, much like our first case who developed drug induced hepatitis. Also, there has been a trend in South Asia (including Nepal) to treat septicemic patients initially with parental drug ceftriaxone. However *B. pseudomallei* is a resistant bug, which is susceptible specifically to drugs like ceftazidime and carbapenem but doesnot respond to ceftriaxone [9, 16]. Its treatment is usually divided into two phases: the first or acute phase in which intravenous ceftazidime or carbapenems with or without trimethoprim-sulfamethoxazole is given for minimum of 14 days and the second or eradication phase in which oral drugs like trimethoprim-sulfamethoxazole with or without doxycycline is given for at least 12 weeks. However, depending upon the clinical responses and the severity of infection, the acute and the eradication phase can be extended for 4 weeks and 20 weeks respectively [16].

The mortality rate of melioidosis is considerably high, which ranges from 19 to 40%. Strikingly, the number may dramatically soar up (> 80%) when septic shock supervenes [17–19]. Therefore, the clinicians ought to be aware of the clinical presentations of melioidosis and the condition should be diagnosed as early as possible. It is of paramount importance to invariably ascertain the travel history and the occupational history of patients with atypical presentation. Apart from travelling to traditionally endemic regions, visiting lately melioidosis-prone countries like India may pose the risk of infection. Also, it becomes mandatory for clinicians to collaborate and discuss aforementioned cases with microbiologists as isolation of *B. pseudomallei* is likely the only means of confirmation.

Additionally, it should be noted that *B. pseudomallei* is quite often confused with other *Burkholderia* species. Therefore, microbiologists need to be suspicious while encountering any gram-negative organisms with safety pin appearance and oxidase positive reaction, which invariably should be further tested. *B. pseudomallei* characteristically produces arginine decarboxylase and forms wrinkled colony within 48 h of incubation unlike other *Burkholderia* species. Further it utilizes glucose and maltose oxidatively, reduces nitrate; and can grow at 42 °C. It is also resistant to aminoglycosides and polymyxin B.

## Conclusion

In Nepal, most of the clinicians are unaware of the clinical presentation of melioidosis; therefore, they often misdiagnose the condition for tuberculosis. Clinicians should suspect melioidosis as a differential diagnosis when any febrile patient with multiple abscesses and predisposing factors like diabetes does not respond to antibiotics easily, especially if the patient has a history of travelling to melioidosis prone area. The disease may manifest in the patient several years after their return back to home, much like our reported cases. Simultaneously, occupational history is equally important as one cannot rule out the possibility of local acquisition. Finally, microbiologists and laboratory technicians should be sufficiently trained so that they do not confound the organism for other *Burkholderia* species. The disease has a high mortality rate; therefore, it should be diagnosed at its earliest possible stages.

## Abbreviations

ATT: Antitubercular therapy
CT: Computed Tomography
ESR: Erythrocyte sedimentation rates
KOH: Potassium hydroxide
TUTH: Tribhuvan University Teaching Hospital
